# Digital-Intelligent Precision Health Management: An Integrative Framework for Chronic Disease Prevention and Control

**DOI:** 10.3390/biomedicines14010223

**Published:** 2026-01-20

**Authors:** Yujia Ma, Dafang Chen, Jin Xie

**Affiliations:** 1 Department of Non-communicable Chronic Disease Control and Prevention, Beijing Center for Disease Prevention and Control, Beijing 100013, China; yujiama@pku.edu.cn; 2 Department of Epidemiology and Health Statistics, School of Public Health, Capital Medical University, Beijing 100069, China; 3 Department of Epidemiology and Biostatistics, School of Public Health, Peking University, Beijing 100191, China; 4 Key Laboratory of Epidemiology of Major Diseases (Peking University), Ministry of Education, Beijing 100191, China

**Keywords:** non-communicable diseases, digital health, artificial intelligence, precision medicine

## Abstract

Non-communicable diseases (NCDs) impose an overwhelming burden on global health systems. Prevailing healthcare for NCDs remains largely hospital-centered, episodic, and reactive, rendering them poorly suited to address the long-term, heterogeneous, and multifactorial nature of NCDs. Rapid advances in digital technologies, artificial intelligence (AI), and precision medicine have catalyzed the development of an integrative framework for digital-intelligent precision health management, characterized by the functional integration of data, models, and decision support. It is best understood as an integrated health management framework operating across three interdependent dimensions. First, it is grounded in multidimensional health-related phenotyping, enabled by continuous digital sensing, wearable and ambient devices, and multi-omics profiling, which together allow for comprehensive, longitudinal characterization of individual health states in real-world settings. Second, it leverages intelligent risk warning and early diagnosis, whereby multimodal data are fused using advanced machine learning algorithms to generate dynamic risk prediction, detect early pathological deviations, and refine disease stratification beyond conventional static models. Third, it culminates in health management under intelligent decision-making, integrating digital twins and AI health agents to support personalized intervention planning, virtual simulation, adaptive optimization, and closed-loop management across the disease continuum. Framed in this way, digital-intelligent precision health management enables a fundamental shift from passive care towards proactive, anticipatory, and individual-centered health management. This Perspectives article synthesizes recent literature from the past three years, critically examines translational and ethical challenges, and outlines future directions for embedding this framework within population health and healthcare systems.

## 1. Introduction

Non-communicable diseases (NCDs) present an extremely severe global health crisis. They account for approximately 41 million deaths annually, which constitutes 75% of total global mortality [1]. The management of NCDs is complex, as they usually require prolonged, and often lifelong, medical intervention and health management. Meanwhile, multimorbidity is prevalent. Yet, the current mainstream health management patterns reveal several systemic and structural challenges when addressing NCDs, which are mainly reflected in the following aspects:

(1) Passive responses and delayed interventions: traditional patterns rely on healthcare providers and exclude patients from decision-making, leading to missed opportunities for early intervention in the subclinical stage, preventing effective prevention and reversal.
(2) Limitations of population-based strategies: current guidelines and public health policies focus on general population-level evidence, which may not suit individual patients, making one-size-fits-all strategies less effective and potentially harmful [2].
(3) Fragmented health data: patient health records are scattered across various systems and institutions, creating gaps in data integration, preventing a complete, continuous health profile, and hindering healthcare providers’ ability to understand a patient’s overall health accurately.
(4) Disconnection in the healthcare service: traditional patterns focus mainly on hospital-based diagnosis and treatment, often neglecting post-discharge rehabilitation and ongoing disease management. This leads to fragmented patient–provider interactions, resulting in poor adherence and suboptimal long-term outcomes.

**Figure 1:**
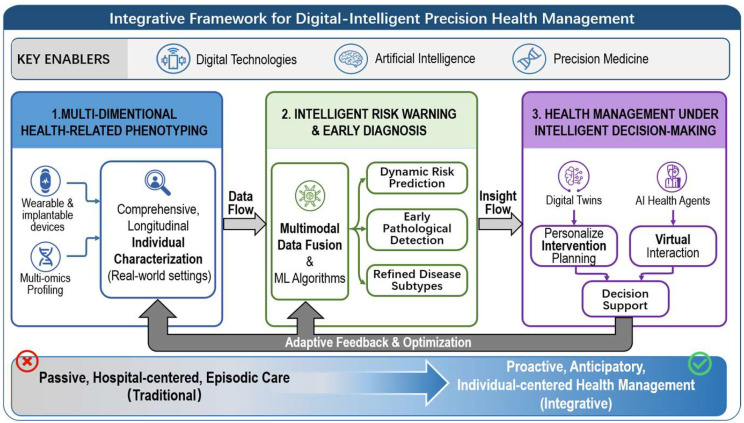
Framework of the digital-intelligent precision health management.

These challenges underscore the urgent need for a new health management paradigm focused on real-time monitoring, proactive risk prediction, and personalized intervention. The emerging global shift towards technology-driven health management, termed digital-intelligent precision health management, is built on three core pillars: multidimensional phenotyping, personalized risk prediction and early diagnosis, and intelligent decision-making for precision intervention (Figure 1). By integrating next-generation technologies (IoT, big data, AI) with advanced biotechnologies (multi-omics), it seeks to transform NCD management. [3,4]. Its vision is a shift from passive, disease-centered care to proactive, personalized health management. The following sections explore the three technological pillars, addressing their applications, challenges, ethical issues, and future implications for NCD prevention and control.

## 2. Data-Driven Cornerstone: A Multidimensional System for Health-Related Phenotyping

The digital-intelligent precision health management framework is built on a real-time, multidimensional data acquisition system that captures the full spectrum of an individual’s health. Integrating external digital sensing with internal molecular technologies, it enables systematic monitoring of both macroscopic physiological signals and microscopic molecular events. This integration forms the data infrastructure essential for personalized risk prediction and intelligent decision-making interventions.

### 2.1. External Digital Sensing from Wearable and Implantable Devices

Digital phenotyping is an innovative approach to health monitoring that leverages smart devices, sensors, and mobile applications to continuously collect real-time data on an individual’s behavioral, psychological, and physiological states [5]. Wearable and implantable devices form the backbone of this digital sensing network, enabling a shift from intermittent monitoring to continuous, real-time data streaming.

In cardiovascular monitoring, single-lead ECG patches, like those in the Apple Watch, enable consumer devices to detect arrhythmic events such as atrial fibrillation. A transformative advancement is wireless blood pressure monitoring via photoplethysmography (PPG), progressing from research to commercial use and potentially revolutionizing hypertension management [3,6]. In respiratory disease management, environmental sensors monitor air quality in real time, identifying triggers and factors contributing to exacerbations, and providing early warnings for patients and clinicians [6]. Continuous glucose monitoring (CGM) systems replace fingerstick tests, offering insights into the impact of food, activity, medication, and stress on blood glucose, enabling clinicians to make data-driven adjustments [7]. Digital phenotyping, powered by multimodal sensors, enables objective mental health assessment by analyzing digital biomarkers from human–computer interactions to create digital phenotypes that reflect emotional states and stress levels [8]. These external sensing technologies continuously gather real-time physiological data, creating dynamic health trajectories for individuals.

### 2.2. Internal Molecular Sensing Through Multi-Omics Technologies

Multi-omics technologies mark a paradigm shift in biomedical research, enabling molecular-level analysis of disease mechanisms and precise characterization of individual heterogeneity. This approach provides key biological insights for precision health management, transforming medical practice from symptom control to personalized, causal management.

The multi-omics framework, rooted in molecular biology’s central dogma, creates a comprehensive molecular ecosystem. Genomics, the foundational layer, identifies disease susceptibility loci and rare genetic variants through genome-wide association studies (GWAS) and whole-exome sequencing. Currently, 15% of approved drugs incorporate pharmacogenomics data [9], and polygenic risk scores are increasingly used in primary prevention for cardiovascular disease and type 2 diabetes (T2D). Epigenomics links genotype and phenotype, showing how environmental factors regulate gene expression through DNA methylation, histone modifications, and chromatin accessibility. Methylation signatures in cell-free DNA hold promise for multi-cancer detection and tissue-of-origin identification [10]. Spatial transcriptomics, when integrated with GWAS data, enables spatially resolved mapping of cells implicated in human diseases and complex traits [11]. Proteomics reveals functional end-products of cellular activity. High-throughput platforms like Olink and SomaScan have identified plasma protein biomarkers in large cohort studies [12,13]. Metabolomics, the final layer of omics, reflects real-time metabolic states shaped by gene–environment interactions. In diabetes, specific amino acids and fatty acid metabolites serve as early biomarkers, with elevated levels up to 12 years before clinical onset [14]. Metagenomics broadens the focus beyond the human host, with emerging evidence linking gut microbiome composition to mental health disorders (via the gut-brain axis), metabolic diseases (through metabolites like short-chain fatty acids), and immune responses to cancer immunotherapy [15,16].

Multidimensional phenotyping underpins the transition from a “one-size-fits-all” model towards personalized and adaptive healthcare, but faces inherent challenges from cross-modal data heterogeneity and noise in continuous monitoring. These issues can be addressed through data standardization using common models (e.g., FHIR, OMOP), combined with entity resolution, feature engineering, and knowledge graph construction to harmonize multi-source data and preserve cross-modal relationships [17]. Scalable data processing platforms and distributed computing frameworks support real-time and batch integration with dynamic model updating, complemented by federated learning for privacy-preserving, cross-institutional model development [18]. In parallel, AI-driven automated data annotation and quality control, together with edge–cloud computation, help reduce sensor noise and latency, ensuring reliable inputs for predictive models [19]. Emerging technologies such as blockchain further enhance data trustworthiness and secure data sharing [20]. Together, these approaches strengthen the robustness and clinical utility of digital twin-based, continuous health management systems.

## 3. Precision Prediction and Detection: Continuous and Dynamic Risk Stratification and Early Diagnosis

Building on multidimensional phenotyping, digital-intelligent precision health management enables disease risk identification and prediction. This section explores how integrating multimodal data with AI algorithms is transforming risk prediction, early diagnosis, and disease subtyping, shifting from data accumulation to knowledge discovery.

### 3.1. Intelligent Early-Warning Framework with Dynamic Risk Prediction Models

Traditional risk prediction models, like the Framingham Risk Score, are static, relying on single-time measurements, and fail to capture dynamic health changes. Intelligent early-warning frameworks based on longitudinal data enable proactive risk prediction and intervention by continuously learning individual baselines and temporal variability. Long short-term memory (LSTM) networks capture long-range dependencies, while Transformer architectures, using self-attention, enable parallel analysis of multivariate time series, revealing complex interactions among physiological parameters [21,22]. By continuously monitoring speech features (e.g., rate and prosody), keyboard patterns (typing speed and errors), social behavior (social media use), and GPS-derived mobility (activity range and social frequency), machine learning algorithms create individualized ‘behavioral fingerprints’ [23]. A nationwide cohort study in South Korea showed that circadian digital phenotypes from wearables and smartphones could predict mood episodes in major depressive disorder patients within three days, with over 90% accuracy [24].

### 3.2. Precision Screening Based on Multimodal Integration and AI Diagnosis

Precision early screening is shifting from single biomarkers to multimodal data and from morphological interpretation to quantitative analysis. In lung cancer screening, multimodal models combining low-dose CT and circulating tumor DNA methylation profiles have improved benign–malignant pulmonary nodule classification accuracy to over 90%, reducing unnecessary surgeries by 89% and treatment delays by 73%, thus mitigating overdiagnosis and delayed diagnosis [25]. AI-based digital pathology quantifies morphological features in stained tissue samples, uncovering key signals that mediate interactions among cancer, stromal, and immune cells [26]. This “computational pathology” approach combines morphological phenotypes with molecular traits, enabling precision prediction and screening, and shifting health management from population-based evidence to personalized, dynamic risk assessment and mechanism-driven disease subtyping.

### 3.3. Novel Subtyping from Data-Driven Disease Reclassification

Disease subtype discovery using multimodal data is driving a shift from symptom-based to mechanism-oriented approaches, underpinned by a rigorous computational framework comprising feature selection, clustering, subtype validation, and biological interpretation [27]. For example, a two-stage DeepCluster model identified three phenogroups of heart failure with preserved ejection fraction, each showing distinct medication responses and prognoses [28]. Recent research integrating genomic and proteomic data has identified molecular markers in heart failure phenogroups, offering new insights for targeted therapy development [29,30]. These data-driven diabetes subgroups mark a new era in management, with treatments tailored to specific subtypes, each linked to distinct comorbidity risk [31]. Data-driven disease subtyping is gaining traction in oncology, with molecular markers like *IDH* mutations and 1p/19q deletions now standard diagnostic criteria for gliomas [32]. Personalized dynamic risk assessments and mechanism-driven subtyping improve intervention timeliness and accuracy, offering insights into disease mechanisms and enabling targeted treatments, shifting from a “one-size-fits-all” to a “right treatment for the right patient at the right time” approach.

## 4. Intelligent Decision-Making: The Ultimate Health Management Powered by Digital Twins and AI

The ultimate goal of digital-intelligent health management is to develop an intelligent system for autonomous decision-making, dynamic optimization, and personalized intervention. This section explores the integration of digital twins and AI in health management, demonstrating how their “virtual simulation–real-time decision-making–dynamic optimization” loop shifts health management from static planning to dynamic, precision execution.

### 4.1. Digital Twin in Simulation and Intervention

A digital twin is a data-driven construct that maps a physical entity to its corresponding virtual model through continuous data transmission; a digital twin in medicine is a digital replication or representation of a physical object, process, or service, augmented with external information such as environmental and social interactions, and continuously fed by a vast amount of dynamic real-time data [33]. By integrating reinforcement learning and multi-agent systems, digital twins can simulate decision-making and social interactions in real-world environments, creating a comprehensive “physiological–psychological–social” model [34]. This approach provides key advantages in the following areas:

(1) Treatment rehearsal: digital twins of coronary arteries, created from patient-specific anatomy and hemodynamic data, enable the simulation of different stent types and implantation strategies, optimizing interventional treatment plans [35,36].
(2) Dynamic drug response prediction: by integrating pharmacogenomics with pharmacokinetic/pharmacodynamic models, digital twins predict drug metabolism and its impact on target pathways. For warfarin dosing, they simulate pharmacokinetic responses in patients with different genotypes over 15 days [37].
(3) Optimal intervention timing: by continuously comparing an individual’s real-time status with their digital twin, critical physiological deviations are identified, enabling timely preventive interventions. In T2D management, this approach has led to remission in 72.7% of patients [38].

### 4.2. AI Health Agents for Autonomous Decision-Making and Personalized Interaction

AI health agents bridge digital twins and individuals, executing health management decisions. These agents integrate four key modules—perception, cognition, decision-making, and interaction—enabling continuous, personalized health management.

(1) Multimodal perception: using a Transformer-based fusion encoder, this module integrates physiological signals, environmental data, and natural language inputs, employing cross-modal attention to establish semantic correlations across data sources [39].
(2) Cognitive reasoning: combining knowledge graphs and deep reinforcement learning, this module enhances healthcare-specific reasoning. The SELF-REFLEX framework enables the agent to assess potential outcomes of decision paths through counterfactual reasoning [40].
(3) Personalized decision-making: using a contextual multi-armed bandit algorithm, this module balances exploring new strategies with exploiting proven interventions [41].
(4) Natural interaction: powered by large language models, this module integrates medical knowledge graphs and empathetic computing to provide health consultations that are both accurate and compassionate [42].

In mental health support, the AI agent analyzes speech and behavior to detect early signs of psychological crises, offering cognitive–behavioral therapy guidance that improves depression symptoms [43].

The integration of digital twins and AI agents is shifting health management from a passive, experience-driven model to a proactive, data-driven paradigm, enhancing efficiency and redefining doctor–patient collaboration and service delivery.

## 5. Future Challenges and Perspectives

The digital-intelligent precision health management framework holds immense potential but faces several challenges in transitioning from concept to large-scale clinical application.

Key technological hurdles—data quality, algorithm efficacy, and system integration—represent the most pressing challenges for real-world implementation. Data heterogeneity, driven by variations in data formats, sampling frequencies, and device accuracy, as well as platform-specific biases in omics data, complicates multi-source integration [44]. The generalizability of multimodal fusion algorithms also remains limited, with current models often underperforming for rare phenotypes and specific populations. In addition, inadequate system interoperability constrains large-scale deployment, as institutional data silos and cross-platform incompatibilities hinder the formation of an integrated health management loop. Solutions such as data standardization, distributed computing frameworks, and AI-driven automated annotation and quality control were discussed in earlier sections.

The key barrier to clinical translation is the lack of evidence from randomized controlled trials, as most digital therapeutic products have yet to prove their clinical effectiveness and cost-efficiency [45]. Thus, clinician and patient acceptance remains a critical issue, with only 32% of clinicians trusting AI-assisted clinical decision-making [46]. Addressing this challenge requires the establishment of clinical evidence-generation frameworks tailored to digital medicine, and a clear articulation of accountability and liability boundaries in human–AI collaborative decision-making.

Ethical challenges are complex, with data privacy and security at significant risk, as cyberattack-related breaches have increased by 239% since 2018 [47]. Algorithmic discrimination remains a concern, as biased training data may cause diagnostic disparities for minorities and women [48]. The widening digital divide also poses risks, as 37% of the global population still lacks regular internet access, exacerbating health disparities [49]. Finally, unclear liability frameworks between manufacturers, institutions, and clinicians hinder accountability in AI-assisted decision-making. These systemic risks require macro-level policy interventions to safeguard health equity and clarify accountability across human–AI collaborative care.

In summary, digital-intelligent precision health management reflects a broader shift from disease-centered care towards health-centered and preventive models. Through the integration of digital twins, AI, and multi-omics technologies, such systems can support continuous monitoring, early warning, and adaptive intervention across the life course. However, realizing this vision requires the convergence of technological innovation with rigorous validation, robust governance, and sustained institutional commitment to ensure safety, equity, and long-term effectiveness in real-world settings.

## Data Availability

No new data were created or analyzed in this study.

## Abbreviations

The following abbreviations are used in this manuscript:

NCDs	Non-communicable diseases
ECG	Electrocardiogram
PPG	Photoplethysmography
COPD	Chronic obstructive pulmonary disease
GWAS	Genome-wide association studies
T2D	Type 2 diabetes
